# Combining artificial neural networks and a marginal structural model to predict the progression from depression to Alzheimer's disease

**DOI:** 10.3389/frdem.2024.1362230

**Published:** 2024-04-05

**Authors:** Sylvester O. Orimaye, Kelly A. Schmidtke

**Affiliations:** ^1^College of Global Population Health, University of Health Sciences and Pharmacy, St. Louis, MO, United States; ^2^College of Arts and Sciences, University of Health Sciences and Pharmacy, St. Louis, MO, United States

**Keywords:** depression, major depressive disorder, Alzheimer's disease, MDD to AD progression, marginal structural model, artificial neural network, predictive model

## Abstract

**Introduction:**

Decades of research in population health have established depression as a likely precursor to Alzheimer's disease. A combination of causal estimates and machine learning methods in artificial intelligence could identify internal and external mediating mechanisms that contribute to the likelihood of progression from depression to Alzheimer's disease.

**Methods:**

We developed an integrated predictive model, combining the marginal structural model and an artificial intelligence predictive model, distinguishing between patients likely to progress from depressive states to Alzheimer's disease better than each model alone.

**Results:**

The integrated predictive model achieved substantial clinical relevance when using the area under the curve measure. It performed better than the traditional statistical method or a single artificial intelligence method alone.

**Discussion:**

The integrated predictive model could form a part of a clinical screening tool that identifies patients who are likely to progress from depression to Alzheimer's disease for early behavioral health interventions. Given the high costs of treating Alzheimer's disease, our model could serve as a cost-effective intervention for the early detection of depression before it progresses to Alzheimer's disease.

## 1 Introduction

**Figure 1:**
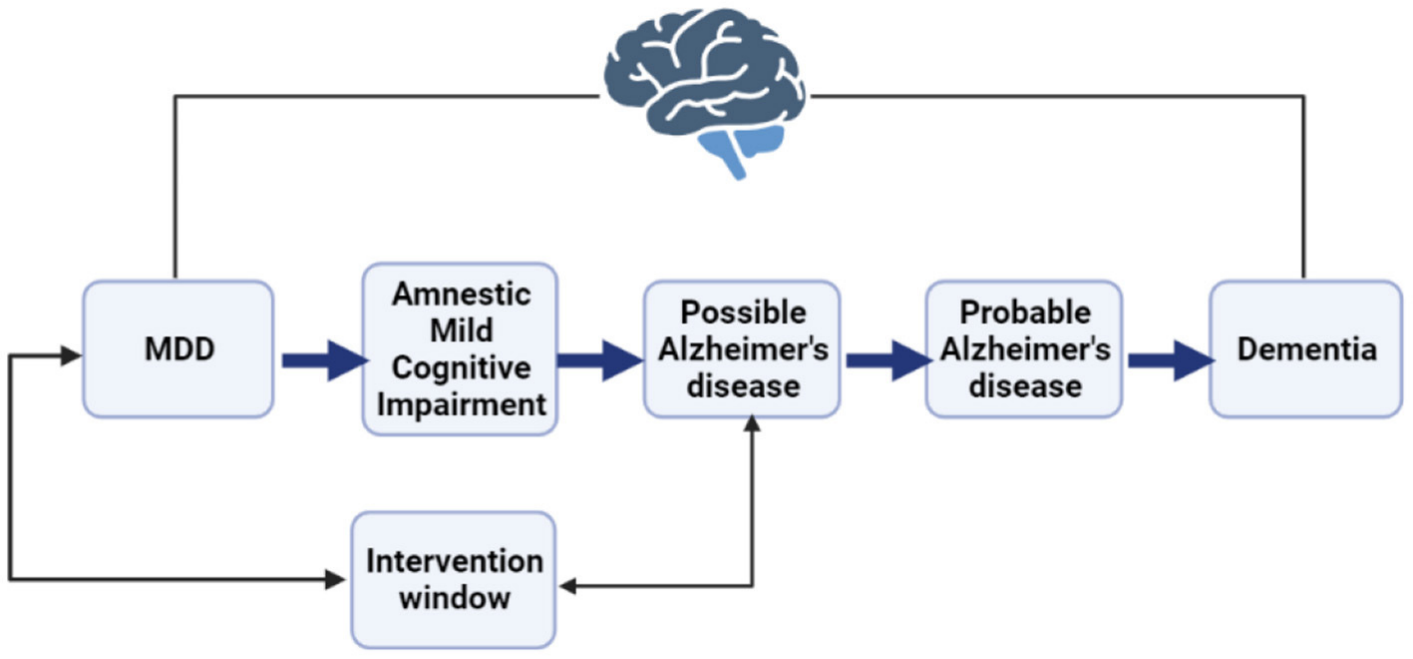
A representation of the major depressive disorder (MDD) to Alzheimer's disease (AD) continuum indicates a potential intervention window to detect patients with the likelihood of progression.

Decades of research in population health have established depression as a likely precursor to Alzheimer's disease (AD) along with internal and external mediating mechanisms (Chen et al., 1999; Jorm, 2001; Kessing and Andersen, 2004; Ownby et al., 2006; Dotson and Beydoun, 2010; Goveas et al., 2011; Köhler et al., 2016; Almeida et al., 2017; Steffens, 2017). Some of these mechanisms are modifiable if intervened early, e.g., deficient serotonin bindings or inaccessible services (Köhler et al., 2016). Further literature shows that depression is highly prevalent among patients with mild cognitive impairment, which may then progress to AD (Figure 1; Barnes et al., 2006; Geda et al., 2006; Dotson and Beydoun, 2010; Goveas et al., 2011; Snowden et al., 2015; Ismail et al., 2017; Ding et al., 2023). Research suggests that depression among patients with mild cognitive disorders substantially accelerates cognitive decline many years before the onset of dementia (Snowden et al., 2015). These findings support the direction of causation (depression then AD) and suggest a window for treatment (Almeida et al., 2017; Steffens, 2017; Dafsari, 2020). Further research could identify those patients most likely to develop AD so that timely treatment could be offered (Thapar et al., 2022). However, investigating each factor individually is resource-intensive. Further understanding how each factor interacts with all the other factors is not feasible without computational assistance. Artificial intelligence models could assist (Orimaye et al., 2020a).

The present study builds on existing literature seeking to predict progression from Major Depressive Disorder (MDD) to AD by integrating artificial intelligence models with more traditional statistical methods, specifically a marginal structural model (MSM) and a Multi-Layer Perceptron (MLP) model (Ding et al., 2023). MSMs are commonly used in healthcare to assess associative relationships and causal relationships between variables in observational data. The use of artificial intelligence models in behavioral health is relatively new (Orimaye et al., 2017; Orimaye and Wong, 2018). While artificial intelligence models can capture complex underlying patterns in healthcare data (Orimaye and Wong, 2018), how those data should be interpreted is not always clear—i.e., the black box concern. In addition, while artificial intelligence training data are adequate for determining associations, they may be limited for identifying causal relationships between variables without a more rigorous methodology. In healthcare, such data are often incomplete, raising concerns regarding whether their findings generalize to the broader population. For instance, commonly used Centers for Medicare and Medicaid Services (CMS) Limited Data Sets (LDS) contain incomplete patient data as, e.g., patients move in and out of enrolment or data are censored. MSMs often apply inverse probability weighting to address such confounding biases. Thus, combining MSMs with an artificial intelligence model may increase prediction power while reducing common concerns about interpretability and generalization (Athey, 2015; Wang and Chen, 2019; Prosperi et al., 2020; Balzer, 2021).

The present study aims to develop an integrated predictive model (IPM), combining MSM and MLP models, that distinguishes between patients likely to progress from depressive states to Alzheimer's disease better than each model alone. In addition to including patient factors (e.g., gender, comorbidities, etc.), we include exogenous factors (e.g., healthcare accessibility) in this model that research already suggests influence progression. Our predictive model has the potential to be at the core of an artificial intelligence-based behavioral analysis and intervention clinical screening tool that identifies patients who are likely to progress from depression to AD for early behavioral health intervention. Using such an integrative approach has previously proved effective for diabetes care provisioning (Kalia et al., 2022). To our knowledge, this is the first time such an integrative approach has been used to predict the progression from depression to AD.

## 2 Materials and methods

### 2.1 Study population/datasets

The primary data sources for our study are the de-identified Medicare inpatient and skilled nursing facility files of the Centers for Medicare and Medicaid Services (CMS) Limited Use Datasets (LDS). The dataset contains 6,567,071 Medicare beneficiary claims from 2012 to 2019, which comprise 5% of the total Medicare beneficiaries. CMS is a federal health care program in the United States that is mainly composed of residents who are 65 and older. From this data set, we identified 282 MDD patients who progressed to AD and a randomly selected equal number of patients with MDD who never progressed to AD between 2012 and 2019.

We used the International Classification of Diseases, Clinical Modification (ICD-CM) coding systems, including ICD-9-CM and ICD-10-CM, to identify depression and AD diagnoses. The ICD-9-CM diagnosis applies to data from 2012 to 2015, and the ICD-10-CM diagnosis applies to data from 2016 to 2019.

Together with the CMS LDS, we used the county-level Health Professional Shortage Areas (HPSA) classification to identify the shortage of health professionals in each patient's county and the 2013 county-level Economic Research Service Rural-Urban Continuum Codes (RUCCs) to classify whether an observation is from a designated rural or urban county (Hardeman et al., 2022; Tan et al., 2022). The Office of Management and Budget uses the RUCC to classify large metros with at least 1 million residents and small metro areas with <1 million residents as urban. Other non-metropolitan regions are classified as rural.

### 2.2 Study variables

#### 2.2.1 Outcome variable

We measured the outcome variable as a binary representation of patients who, between 2012 and 2019, made at least one claim for depression and later made at least one claim for AD. We excluded observations where claiming an AD diagnosis precedes a depression diagnosis. Thus, each patient's MDD diagnosis must precede the AD diagnosis within the longitudinal 8-year period. Further, the underlying unique pattern for predicting a future outcome (progressed to AD or not) is captured in the variability of the predicting variables, many of which increase or decrease over a longitudinal period.

#### 2.2.2 Predictor variables

Predictor variables include demographic variables: *age, gender, race*, and *rural-urban* location. The patient's location was included because there is growing evidence of increasing disparities in the prevalence of AD between the urban and rural parts of the United States (Bradford et al., 2009; Mehta, 2017; Zissimopoulos et al., 2018; Jack et al., 2019; Orimaye et al., 2020b). Additional predictors include the *HPSA* classification that captures counties with shortages of health professionals for access to early and preventive behavioral health services, including mental and behavioral health practitioners, dentists, and primary care providers. The HPSA classification includes a whole shortage area (the entire county is underserved with health professionals), a partly shortage area (the county has some health professionals), and no shortage (the county has substantial health professionals). Research has shown that accessibility to early detection through professional healthcare services provides effective treatment strategies for preventing AD (Eichler et al., 2015; Orimaye et al., 2017, 2020a; Dafsari, 2020).

Other variables include Medicare *utilization days count after MDD diagnosis* through inpatient hospital admission or residence at a skilled nursing facility, any *comorbidity* diagnoses secondary to or co-occurring with the primary major depressive disorder (MDD) diagnosis, the *count of claims for* MDD diagnosis indicating the probable number of patient-provider visits for pharmacological or non-pharmacological intervention, and a *follow-up visit* variable that indicates whether patients made subsequent claims or visits after their first MDD diagnosis.

### 2.3 Assessment and diagnosis of depression

We operationalized MDD as clinical depression that includes all three severity levels of depression (mild, moderate, and severe) for single (F.32) and recurrent (F.33), including recurrent brief depressive episodes (Hasin et al., 2018). We consider 296.2X for ICD-9-CM and the F.32.X for ICD-10-CM (Association, 2015). According to the DSM-5, MDD is characterized by a minimum of five depressive mood symptoms presenting as feelings of sadness, emptiness, hopelessness, tremendous weight loss or daily bidirectional changes in appetite, insomnia or hypersomnia, agitation, fatigue, feelings of worthlessness, reduced cognitive or executive functions, and thoughts of suicide.

### 2.4 Assessment and diagnosis of Alzheimer's disease

Using the ICD codes in the CMS LDS, we identified AD with ICD-9-CM code 331.0 and ICD-10-CM codes G30.0, G30.1, G30.8, and G30.9 (Sachdev et al., 2014). Based on the DSM-5, AD is a progressive neurodegenerative disease that causes the death of nerve cells in various parts of the brain, mainly in the frontotemporal region. This loss of function results in a decline of cognitive abilities such as memory and language.

### 2.5 Multi-layer perceptron

We learned the underlying representation of the different levels of abstraction of the covariates and predictors using an efficient backward propagation (backpropagation) method for the Multi-Layer Perceptron (MLP) deep learning algorithm (Zhou et al., 2021). The backpropagation method uses the chain rule, enabling the MLP to systematically understand the underlying structure in reverse order, beginning from the output layer of the network (outcome variable) to the input layer (covariates and predictors). Thus, the MLP considers the complex underlying representation of the observational data and the contextual interaction among the covariates and predictors, often challenging to identify by standard regression models and incredibly difficult to calculate manually. The underlying abstraction of the data is captured as the weights connecting several internal layers of the predictor and covariate structures. A unique underlying weight structure adequately predicts the progression from depression to AD. Similarly, the control group, non-progressive depression (no progression from depression to AD), has an underlying weight structure, which is inherently different from the progression group.

We performed a grid search to identify optimal values for the MLP. Parameter values include the rectified linear unit (ReLU) activation function, the limited-memory Broyden–Fletcher–Goldfarb–Shanno (lbfgs) solver, an alpha of 1e-8, tolerance level of 1e-3, learning rate initialization of 0.1, hidden layers of 6, max iteration of 1,200, and a random state of 4. ReLU is known for its exemplary performance in computing the predicted probabilities for the underlying weight structure (Hara and Saito, 2015). Also, the lbfgs is known for its efficiency on small to medium datasets, as observed in our training set. Finally, we trained the MLP model on 80% of the dataset and tested it with the remaining 20%.

**Figure 2:**
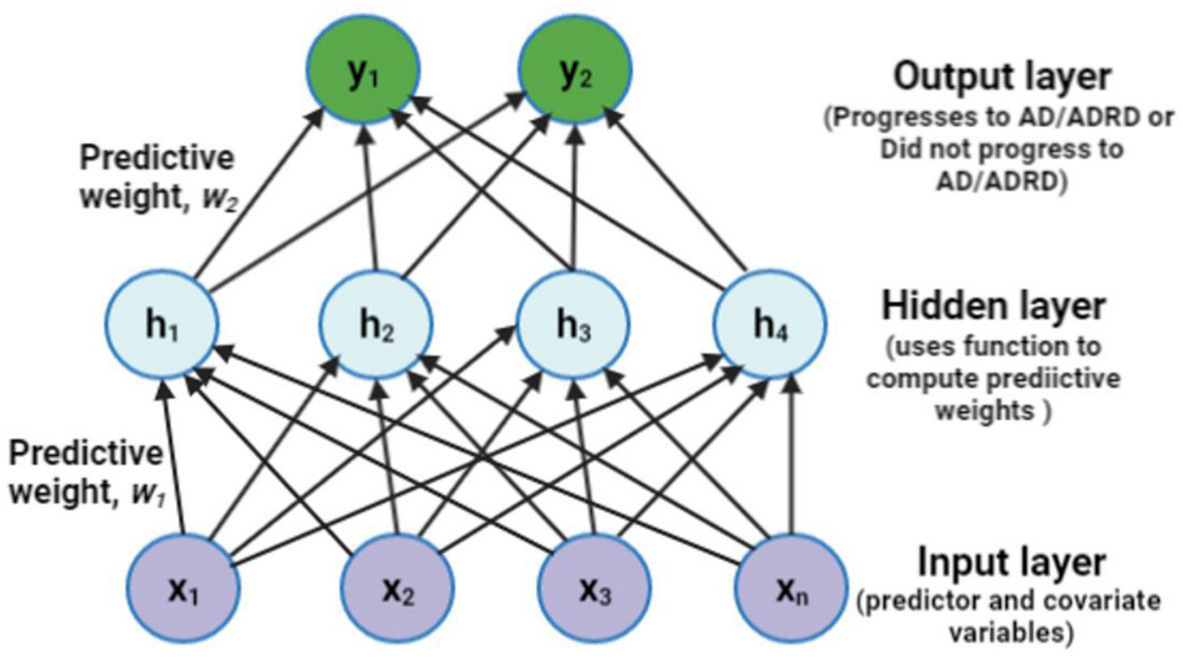
A sample representation of the proposed Multi-Layer Perceptron (MLP) with one hidden layer of four units. ADRD means Alzheimer's disease (AD) and related dementia, often used interchangeably with AD alone.

The proposed fully connected structural representation of the observational data using the MLP architecture is presented in this section. With the predictors and covariates fed as inputs to the network structure, the hidden layer helps compute the weights using a specified activation function. The weights capture the underlying pattern within the data. The MLP then classifies the output as one of the outcome categories. Figure 2 depicts a simple three-layer structure of an MLP model.

### 2.6 Marginal structural model

MSM is a class of causal models on observational data, which accounts for time-varying confounders when examining the effect of a time-dependent exposure. Its use has been encouraged to eliminate bias in observational data (Athey, 2015; Prosperi et al., 2020).

**Figure 3:**
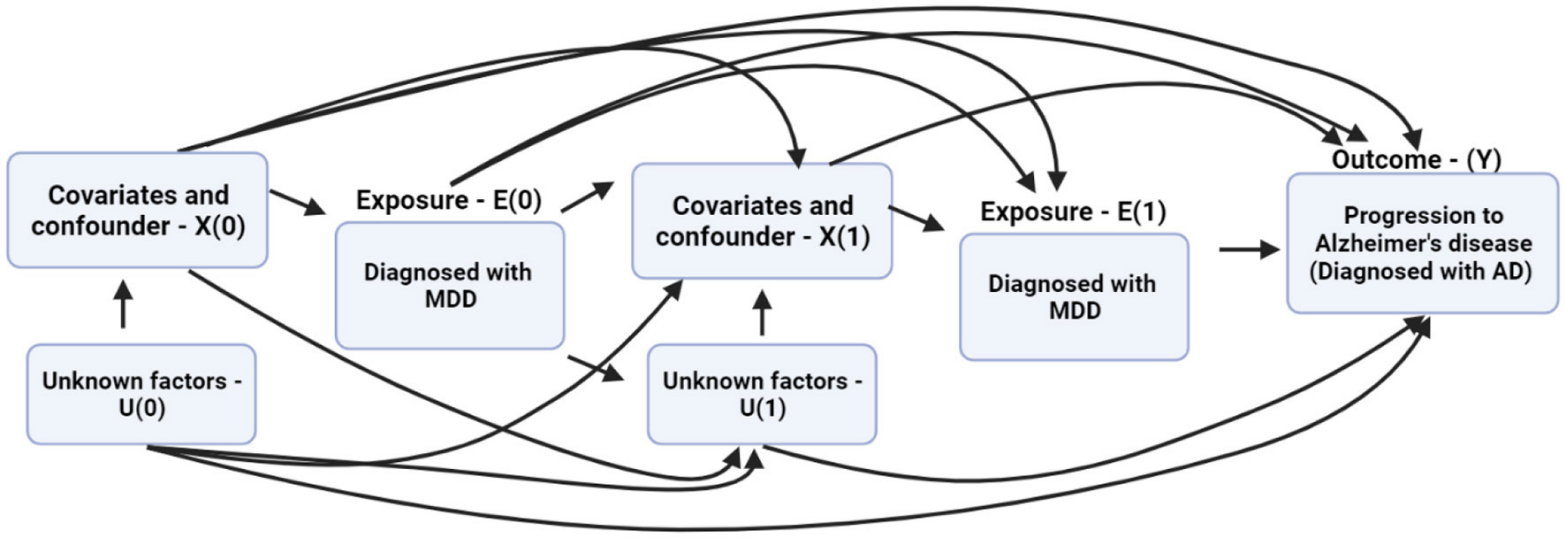
The directed acyclic graph (DAG) describes the relationship between the exposure, covariates, time-varying predictors, and progression from major depressive disorder (MDD) to Alzheimer's disease (AD).

Thus, we present the causal model as a directed acyclic graph (DAG). The DAG indicates the time-varying covariates, confounders, and the underlying connection with MDD diagnosis and the risk of progression to AD. In the DAG, *X* indicates the factors affecting the progression from MDD to AD, *E* denotes the exposure for having been diagnosed with MDD, *U* is the unknown factor(s) affecting the outcome, and *Y* is the outcome, which is the progression from MDD to AD. The DAG shows that the probability of being diagnosed with MDD at E(0) is conditioned on covariates and confounding factors X(0) in the data. E(1) and X(1) are conditioned on E(0) and X(0). The inverse of these conditional probabilities of receiving exposure E(1) given E(0) and X(0) are referred to as the inverse probability of treatment weights (IPTW; Austin, 2016). *A priori*, the IPTW for the MSM is calculated using the following equation:


(1) $$\begin{array}{l} IPTW=\;\overset{T}{\underset{t=0}{\prod }}\frac{P\left(E_{t}|E_{t-1},X_{0}\right)}{P\left(E_{t}|E_{t-1},X_{0},X_{t-1}\right)} \end{array}$$

where *t* indicates the time lag in years for *T* number of years with *t* = 0 as the baseline year and *t*−1 as the 1-year time lag, *E*_*t*_ indicates the MDD diagnosis at a particular time or time lag in years. X is a set of time-varying and non-time-varying confounders. The conditional probabilities for the numerator and denominator were computed using the predicted probabilities from a logistic regression model that controls for the non-time-varying and time-varying confounders. Finally, the IPTW computes a stabilized weight that controls for time-varying confounding variables. It provides better causal estimates on observational data. Figure 3 depicts a simple MSM.

### 2.7 Integrated predictive model (IPM)

Our integrated model combines the MSM and MLP models using an ensemble learning method called the Gradient Boosting Classier (GBC; Wade, 2020). This method uses an additive model to reflect the contribution of each model. Hyperparameter tuning was performed to control overfitting by introducing randomness to the model using a 60% subsample of the training set for each boosting iteration without replacement. Thus, the parameters for the GBC include random_state = 28, n_estimators = 100, learningrate = 0.01, max_depth = 3, and subsample = 0.6. Also, each model in the ensemble model used the same training data. The integrated prediction for progression from MDD to AD is computed using Equation 2 as follows:


(2) $$\begin{array}{l} IPM_{i}=\left[pANN_{i},\;pMSM_{i}\right] \end{array}$$

where *IPM*_*i*_ is the integrated prediction model for the *i*-th observation, *pANN*_*i*_ indicates the MLP model prediction for the *i*-th observation, and *pMSM*_*i*_ is the MSM prediction for the *i*-th observation. Thus, we used the IPM to classify unseen observations likely to progress from MDD to AD. The IPM weighs the predictions from the *pANN* and *pMSM* models and effectively integrates them as features for the final predictive outcome. The predictive features from each model represent varying aspects of the underlying patterns of the data. More importantly, the MSM features capture marginal relationships between the variables, while the MLP features capture more complex, non-linear patterns from the data. Therefore, the IPM leverages the diverse feature representations to make the final predictions.

Further, we evaluated the model using the Area Under the Curve (AUC), accuracy, precision, recall, and the f1-score (Jiao, 2016; Carrington et al., 2022).

### 2.8 Sensitivity analysis

We performed sensitivity analysis (SA) as part of the safeguards for prediction modeling and explainable artificial intelligence models from observational data (Smith, 2021). Specifically, we demonstrated the robustness and precision of the IPM using external validation techniques (Peters and Bühlmann, 2016). Thus, we deliberately distorted the original dataset by randomly replacing 40% of the data with missing values across all the predictors. Further, we used the Markov Chain Monte Carlo multiple imputation technique to impute the missing values using the original data's predictive distribution (multivariate normal distribution; Rezvan and Lee, 2015). We then evaluated the IPM on the imputed dataset and compared findings to the original dataset.

### 2.9 Statistical analysis and machine learning platforms

To show the similarity between the characteristics of patients who *remained MDD* and *progressed to AD*, we conducted the chi-square test of independence on all the categorical variables (*age, gender, race, rural-urban, HPSA, comorbidity*, and *follow-up visit*). Further, we performed a two-sided independent sample *t*-test on all numerical variables (*follow-up visit and count of claims for MDD*). We conducted *t*-tests, chi-square tests, and logistic regression models for computing the IPTW stabilized weights (Equation 1) using the Statistical Analysis Software (SAS) version 9.4 on SAS Studio. All statistical tests were performed at a 95% confidence interval (CI). We also used Python 3.12.1 to develop the MSM with the Weighted Least Squares (WLS) estimation function of the *statsmodels* module version 0.15.0. The WLS function takes the IPTW as weights to fit the MSM and generates the predictive probabilities. Finally, we used the Scikit-learn version 1.1 Python machine learning module to implement the MLP model and the Gradient Boosting Classifier that performed the IPM. Note that 80% of the patients per group were used as the training set. There were 282 beneficiaries per group (remained MDD vs. progressed to AD), which equates to ~226 patients per group for training and ~56 patients for testing. The exact proportions were used across the IPM, MSM, and MLP.

## 3 Results

### 3.1 Sample characteristics

Table 1 shows the descriptive characteristics of the samples used in developing our model. A total of 282 unique patients met the definition of the outcome variable by progressing from MDD to AD diagnosis. A randomly selected group of 282 patients who remained MDD as of 2019 was used as a control group. Patient demographics were broadly similar across groups par the statistical significance tests at 95% CI. Descriptively, there were more older patients who progressed to AD compared to those who remained MDDs. Both groups contained a higher percentage of female than male patients (remained MDD 59.22% and progressed to AD 65.96%). White was the most common ethnicity in both groups (remained MDD 85.46% and progressed to AD 86.88%) groups. Most patients lived in rural locations (remained MDD 82.62% and progressed to AD 72.34%), experiencing partial healthcare shortages (remained MDD 56.03% and progressed to AD 62.77%). Both groups utilized healthcare services with similar frequencies (remained MDD 9.65 days and progressed to AD 10.90 days).

**Table 1:** Descriptive characteristics of the sample.

**Predictors**	**Remained MDD (*****N*** = **282)**		**Progressed to AD (*****N*** = **282)**	
	***N***	**%**	***N***	**%**
**Age** ^*^				
<65	117	41.49	32	11.35
65–69	17	6.03	30	10.64
70–74	6	2.13	44	15.6
75–79	100	35.46	66	23.40
80–84	28	9.93	62	21.99
>84	14	4.96	48	17.02
**Gender** ^a^				
Male	115	40.78	96	34.04
Female	167	59.22	186	65.96
**Race** ^a^				
White	241	85.46	245	86.88
Black	29	10.28	27	9.57
Others	12	4.26	10	3.55
**Rural-urban** ^*^				
Rural	233	82.62	204	72.34
Urban	49	17.38	78	27.66
**HPSA** ^a^				
None	30	10.64	32	11.35
Whole	94	33.33	73	25.89
Partly	158	56.03	177	62.77
**Comorbidity** ^a^				
None	78	27.66	64	22.70
One or more	204	72.34	218	77.30
**Follow-up visit** ^a^				
At least two visits	69	24.47	66	23.40
No follow-up visit	213	75.53	216	76.60
	**Mean** ±**St. dev**			
**Utilization days count after MDD diagnosis**	9.65 ± 9.55		10.90 ± 8.39	
**Count of claims for MDD** ^**a**^	1.78 ± 3.03		1.58 ± 2.63	

^*^Statistical significance difference observed between “Remained MDD” and “Progressed to AD” at p <0.05.

^**a**^No statistical significance observed.

### 3.2 Model performance and clinical relevance

**Figure 4:**
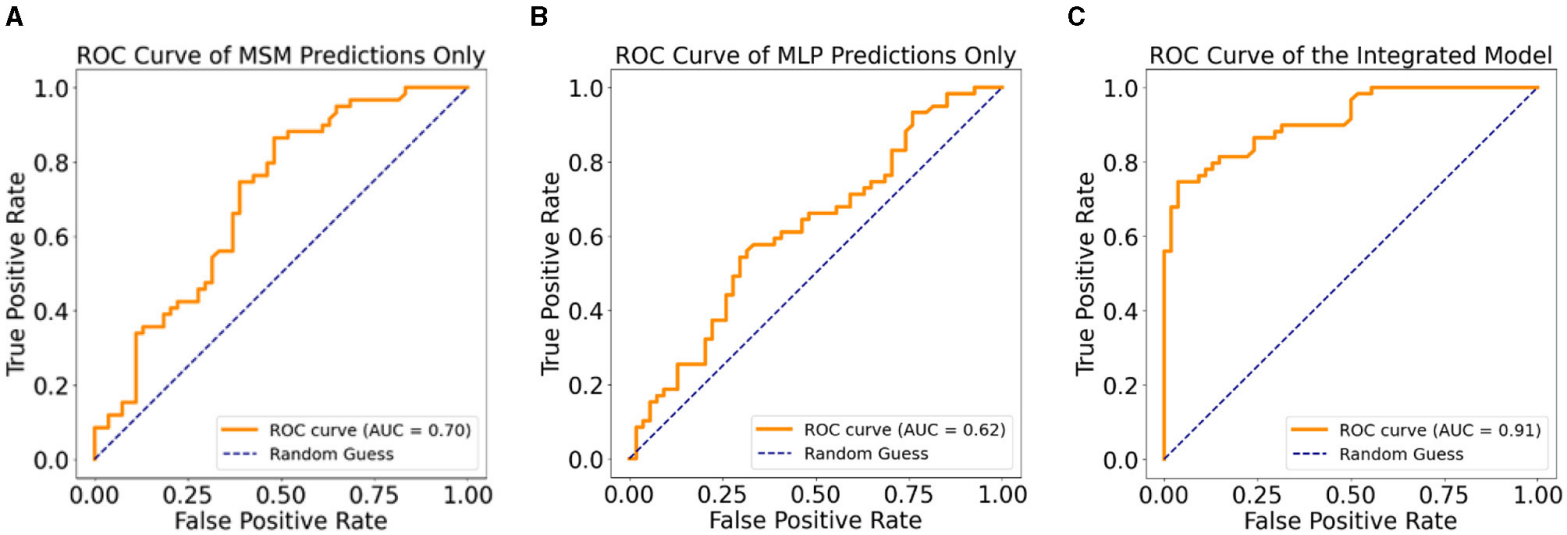
Clinical relevance with ROC curves, **(A)** ROC curve for the MSM, **(B)** ROC curve for the MLP, **(C)** ROC curve for the integrated predictive model.

Table 2 shows the performance of the MSM, MLP, and IPM on 20% held-out test data. In terms of predicting MDD patients who progressed to AD, the MSM outperformed the MLP across all the statistical measures except precision. However, the IPM showed improved model performance across all the statistical measures compared to the MSM or MLP. We observed that the IPM showed a higher precision in detecting patients who remained MDD than those who progressed to AD. Figure 4 shows the graphical representation of the area under the curve (AUC) measure of clinical relevance, depicting the differences between the predictive effectiveness of the MSM, MLP, and IPM. Only the IPM achieved substantial clinical relevance when considering the AUC measure above 80% (Verbakel et al., 2020; Carrington et al., 2022).

**Table 2:** Model performance for predicting progression from depression to AD on a smaller dataset.

**MSM**			**MLP**		**IPM (MSM** + **MLP)**	
**Statistical measures**	**Remained MDD**	**Progressed to AD**	**Remained MDD**	**Progressed to AD**	**Remained MDD**	**Progressed to AD**
Precision	0.66	0.67	0.58	0.66	0.84	**0.71**
Recall	0.61	**0.71**	0.70	**0.53**	0.59	**0.90**
F1-score	0.63	**0.69**	0.63	**0.58**	0.70	**0.79**
Accuracy	0.66		0.62		**0.75**	
AUC	0.70 (CI: 0.61 – 0.80)		0.68 (CI: 0.51 – 0.72)		**0.91** (CI: 0.85 – 0.97)	

Bold values highlight differences in the performance of the three models.

### 3.3 Influencing variables in the IPM

We evaluated the trained models to identify influencing features and their significant contributions to the IPM. Table 3 shows the ranking of features by importance to the ensemble model. We ranked the MSM features using the marginal structural model's weighted least square regression coefficients. Additionally, we ranked the MLP features by computing the feature importance based on the sum of absolute weights for each input feature connected to the first hidden layer of the neural network. Significant coefficients and weights were identified at *p* < 0.05. In determining significant variables, the results of the MSM-fitted model generate the coefficients and the corresponding *p*-values. However, for computing *p*-values for the MLP, we used an independent *t*-test to reject the null hypothesis that the mean of the feature weights is zero.

**Table 3:** Ranked features by importance to the ensemble model.

**Rank #**	**MSM features**	**MSM coefficient**	**MLP features**	**MLP abs. weight**
1	Comorbidity^*^	0.145	Comorbidity	4.600
2	Rural-urban^*^	0.142	Follow-up visit	4.303
3	Age^*^	0.089	HPSA	4.234
4	HPSA	0.036	Rural-urban	3.731
5	Gender	0.015	Gender	3.453
6	Utilization days^*^	0.005	# of MDD diagnosis	3.417
7	Follow-up visit	0.001	Race^*^	2.874
8	# of MDD diagnosis	−0.005	Age	2.854
9	Race	−0.030	Utilization days	1.279

^*^p < 0.05.

Four variables significantly influence the MSM model, with three of the four ranked as the top three features. They include, in order, any *comorbidity* diagnoses secondary to or co-occurring with MDD, residing in a *rural location, age*, and Medicare *utilization days count after MDD diagnosis*. On the other hand, *race* is the only significant feature in the MLP model, but it contains two of the four MSM top-ranked features in its top four ranked features (*comorbidity* and *rural location*). Thus, we show that the MSM features compensate for the bias in the IPM as they capture the underlying patterns in the data.

### 3.4 Error analysis

We performed an error analysis for the three models using the respective confusion matrices generated on the test data alone. Again, each model was trained on a separate training set independent of the test set. We used each model's confusion matrix to compute the models' overall precision, recall (sensitivity), and specificity. The IPM shows 89.8% precision (53 true positives and 6 false positives), showing a minimal rate of false positives compared to MSM's 71.2% (42 true positives and 17 false positives) and MLP's 52.5% (31 true positives and 28 false positives). However, the IPM has a slightly low recall, 75.7%, but 86.0% specificity compared to its precision value. MSM and MLP showed the same recall values at 66.7 and 66.0%, but the MSM has a moderately higher specificity (66%) than the MLP (57.6%). Thus, the IPM shows evidence of a low rate of false positives (wrong predictions) among patients who remained MDD while correctly identifying patients who are likely to progress to AD the majority of the time.

### 3.5 Results of the sensitivity analyses

**Figure 5:**
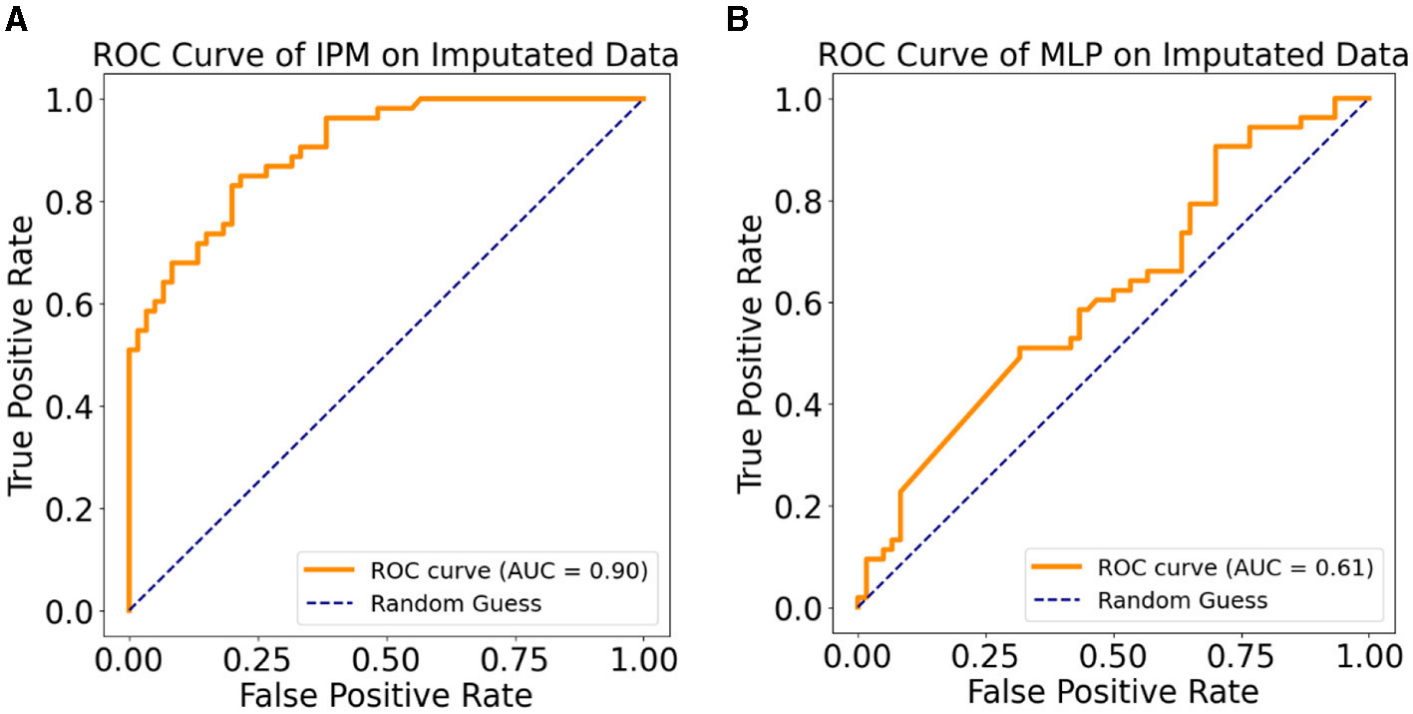
**(A)** ROC curve of the IPM on multiple imputation data. **(B)** ROC curve of the MLP model on multiple imputation data.

Tables 4A, B show the results of the Sensitivity Analysis (SA) performed on the imputed data of the original sample using the IPM and an MLP-only model. The IPM maintained its effectiveness and clinical relevance in predicting patients progressing to AD. The AUC clinical relevance measure remained above 80% (Figure 5A). Also, the IPM showed a high precision value of 82% despite the distortion in the data.

**Table 4:** (A, B) Model performance from the sensitivity analysis using imputed data (*N* = 564).

**Statistical measures**	**Remained MDD**	**Progressed to AD**
**(A) Sensitivity analysis—MLP and MSM**		
Precision	0.76	**0.82**
Recall	0.87	**0.68**
F1-score	0.81	**0.74**
Accuracy	**0.78**	
AUC	**0.90** (CI: 0.84 – 0.96)	
**(B) Sensitivity analysis—without MSM**		
Precision	0.57	0.49
Recall	0.40	0.66
F1-score	0.47	0.56
Accuracy	0.52	
AUC	0.61 (CI: 0.51 – 0.72)	

Bold values highlight differences in the performance of the three models.

Furthermore, Figures 5A, B demonstrate how the MSM helps with bias removal in two ways: first, by modeling with the IPTW for estimating treatment effects. The MSM features comprise IPTW, which computes a stabilized weight that controls for time-varying confounding variables. We emphasize that using IPTW has been proven to eliminate bias compared to using ordinary variables as feature representation (Athey, 2015; Prosperi et al., 2020). Second, the sensitivity analysis confirms the effect of the estimated treatment effect on the IPM by removing the IPTW from the model and testing on the imputed dataset. Compared to the sensitivity analysis results with the IPM, the MLP-only model showed a lower AUC (Figure 5B). This shows the benefit of the removed IPTW features as a bias-removing mechanism.

## 4 Discussion

In this study, we demonstrated the effectiveness of a clinically relevant integrated predictive model (IPM) over a marginal structural model (MSM) alone or the Multi-Layer Perceptron (MLP) model alone. Our findings suggest that the IPM can combine the strength of the other models to differentiate patients diagnosed with major depressive disorder (MDD) who are likely to progress to Alzheimer's disorder (AD) from those who do not. Below, we briefly review the relevance of these findings in light of previous research and highlight the potential benefits of future clinical practice.

The effectiveness of using artificial neural network models, like our MLP algorithm, in predicting health outcomes is an attractive idea (Orimaye and Wong, 2018; Zhou et al., 2021). However, the strength of an MLP predictive model is better realized on large datasets. In this study, the temporal gap between MDD and AD diagnosis limits the number of patients that meet the definition of the outcome variable (*N* = 282). Nonetheless, the statistical measure of precision demonstrated by the MLP model (Table 2) is on par with the MSM (67 vs. 66%). Further, the AUC measure of clinical relevance for the MLP model is only two points lower than the traditional MSM model (68 vs. 70%).

Clinical support for combining artificial intelligence models with other statistical models requires careful analysis and validation across different statistical measures. Previous research focused on diabetes (Kalia et al., 2022). Our study focused on predicting AD. Future studies may develop models for other diseases. Recent studies have attested to the effectiveness of using an ensemble of models to create an integrated predictive model (MacKay et al., 2021). In particular, the gradient-boosting classification algorithm used in combining the MSM and MLP predictive probabilities has a track record of reliability in healthcare data (Zhang et al., 2019; Rufo et al., 2021). The literature attributes the effectiveness of gradient-boosting classifiers to the careful combination of several weak decision tree learning methods to create a more robust decision tree with better predictive accuracy (Wade, 2020).

The precision of the IPM in predicting progression from depression to AD supersedes that of MSM and MLP by at least 5 points. Effective classifiers tend to have similar precision and recall values. We believe that the limited sample size for the outcome can explain the gap between the precision and recall measures for the IPM (71 vs. 90%). Nevertheless, the F1-score, accuracy, and AUC statistical measures for the IPM demonstrated robust predictive performance compared to the MSM or MLP alone. More importantly, findings from the sensitivity analysis further attest to the robustness of the IPM model. Despite the distortion introduced to the data, the IPM showed a substantial AUC close to the AUC measured on the original dataset (Table 4A). With only 60% of the distorted sensitivity analysis data maintaining an actual discriminant pattern, we believe the IPM model correctly emphasizes precision using only the portion of the data exhibiting discriminant features.

The IPM could form a part of a clinical intervention screening tool that identifies patients likely to progress from MDD to AD for early behavioral health interventions. In addition to other freely available datasets used in this study, we anticipate future research sponsorship by the CMS or one of its agencies such that access to the entire CMS Medicare data would be free for large-scale model development. Using the model on electronic medical records in real-time could save health systems and CMS costs by identifying patients potentially in need of early behavioral health intervention before they progress to AD. Given the high costs of treating AD (Lynch, 2018), our model could be cost-effective. However, how this tool is sustained across time and what interventions are offered are matters for future research.

This present study is not without limitations. First, the CMS LDS dataset used in this study has inherent limitations, which are popularly discussed in the literature (Lee et al., 2019; MacKay et al., 2021; Velasquez and Orav, 2023). The CMS LDS dataset does not provide the clinical diagnostic criteria and instruments for any disease outcomes (MDD, AD, and other comorbidities). Second, the lack of continuous enrollment for some eligible Medicare beneficiaries creates censoring problems that are challenging to capture. For instance, the data failed to capture why some patients diagnosed with MDD did not make subsequent claims or visits after their first diagnosis. Whether the patients stopped because an intervention (treatment) worked after their first diagnosis or they stopped because they did not want to pursue therapy is unclear. Third, the control group was never matched using statistical methods such as propensity score matching (Caliendo, 2008). Instead, we randomly selected an equal number of MDD patients. In addition to the many practical limitations for creating a set of balanced covariates across demographics such as age and race in healthcare research (Reiffel, 2020), a matched set of observations will likely limit the variability of the model, thereby reducing the generalizability. Also, the intention of this study was not to estimate the effect of an intervention as often required in targeted quasi-experimental studies on observational data (Reiffel, 2020). Fourth, unlike the MSM, it is still challenging to describe the effectiveness of the learned weights of the artificial neural networks relative to the combined model. Finally, the CMS LDS dataset used in this study comprises only 5% of the total inpatient and skilled nursing facilities data. The dataset excludes outpatients and carrier files, which could provide additional observations relating to MDD patients progressing to AD. This explains the small sample size of 282 patients who progressed to AD over 8 years. Therefore, future work should endeavor to train the predictive model on a combination of inpatient, outpatient, and skilled nursing facilities and the carrier files data to improve the model's generalizability.

## Data availability statement

Publicly available datasets were analyzed in this study. This data can be found at: The CMS Limited Datasets were analyzed in this study. Information about the CMS LDS data can be found at: https://www.cms.gov/data-research/files-for-order/limited-data-set-lds-files. The county-level Health Professional Shortage Areas (HPSA) classification can be found at: https://bhw.hrsa.gov/workforce-shortage-areas/shortage-designation#hpsas. The 2013 county-level Economic Research Service Rural-Urban Continuum Codes (RUCCs) can be found at: https://www.ers.usda.gov/data-products/rural-urban-continuum-codes/.

## Ethics statement

The studies involving humans were approved by East Tennessee State University. The studies were conducted in accordance with the local legislation and institutional requirements. Written informed consent for participation was not required from the participants or the participants' legal guardians/next of kin in accordance with the national legislation and institutional requirements.

## Author contributions

SO: Writing – review & editing, Writing – original draft, Visualization, Supervision, Software, Methodology, Investigation, Funding acquisition, Formal analysis, Data curation, Conceptualization. KS: Writing – review & editing, Supervision, Resources, Project administration, Investigation, Funding acquisition.
